# Web-Based Exercise Interventions for Children with Neurodevelopmental Disorders

**DOI:** 10.3390/pediatric15010010

**Published:** 2023-02-09

**Authors:** Natsumi Ikezawa, Ryo Yoshihara, Masahiro Kitamura, Ayami Osumi, Yuji Kanejima, Kodai Ishihara, Kazuhiro P. Izawa

**Affiliations:** 1 Department of Health Science, Faculty of Medicine, Kobe University, Kobe 654-0142, Japan; 2 Cardiovascular Stroke Renal Project (CRP), Kobe 654-0142, Japan; 3 Department of Public Health, Graduate School of Health Sciences, Kobe University, Kobe 654-0142, Japan; 4 Department of Rehabilitation, Kobe City Medical Center General Hospital, Kobe 650-0047, Japan; 5 School of Physical Therapy, Faculty of Rehabilitation, Reiwa Health Sciences University, Fukuoka 811-0213, Japan; 6 Department of Rehabilitation, Sakakibara Heart Institute of Okayama, Okayama 700-0804, Japan

**Keywords:** web-based exercise intervention, children, neurodevelopmental disorders, systematic review

## Abstract

Various studies have shown the effectiveness of motor interventions for children with neurodevelopmental disorders (NDDs). Web-based interventions may provide an opportunity for remote access to effective interventions with less burden on therapists. This systematic review aimed to examine the effects of web-based exercise interventions for children with NDDs. We searched PubMed for relevant articles published in English since 1994 and included intervention studies focusing on NDDs in children aged ≤18 years, who received web-based exercise interventions. We categorized the extracted information by outcome measure and intervention type and assessed the risk of bias of the included studies. We selected five articles whose subjects had autism spectrum disorder (ASD), attention deficit hyperactivity disorder (ADHD), and developmental coordination disorder (DCD). The exercise interventions used active video games, a Zoom-based intervention, and a WhatsApp-based intervention. Three papers showed improvements in physical activity, motor function, and executive function, whereas two papers on DCD showed no improvements in motor coordination or physical activity. Web-based exercise intervention for children with ASD and ADHD may improve their motor function, executive function, and physical activity rather than for children with NDDs. An intervention may be more effective when the content of the intervention is based on objectives and symptoms, when guidance is provided by specialists, or when sufficient explanation and support are provided to parents. However, more research is needed to statistically evaluate the effectiveness of web-based exercise interventions for children with NDDs.

## 1. Introduction

Neurodevelopmental disorders (NDDs) are a group of disorders that appear early in development and are characterized by impairments in cognitive function, motor function, verbal communication, social skills, and behaviors [1]. NDDs include autism spectrum disorder (ASD), attention deficit hyperactivity disorder (ADHD), specific learning disorder (LD), intellectual disability (ID), motor disorder (including developmental coordination disorder [DCD] and stereotypic movement disorder), communication disorder, and tic disorder (including Tourette’s disorder) [2]. The prevalence of NDDs differs considerably from country to country, and many patients with NDDs have two or more comorbid disorders [3]. Simonoff et al. [4] reported that seven out of ten people with ASD have no less than one concomitant NDD or psychiatric disease. In addition, there is evidence that the effects of NDDs remain for a lifetime in many people [3]. Furthermore, although it is not included in the diagnostic criteria, motor dysfunction is often comorbid with ASD [5], and Ohara et al. [5] found a correlation between motor skills and social skills in patients with ASD.

Several studies have shown the effectiveness of exercise interventions for children with NDDs [6,7]. Healy et al. [6] reported that exercise intervention for children with ASD improved their motor skills, social functioning, and muscular strength and endurance. Cerrillo-Urbina et al. [7] reported that aerobic exercise intervention for children with ADHD was useful in relieving symptoms, such as inattention, hyperactivity, impulsivity, anxiety, executive dysfunction, and social disorders. However, the limited number of therapists and geographical conditions reduce opportunities for non-drug interventions for children with NDDs [8]. In addition, the COVID-19 pandemic has affected the availability of many services for children with NDDs [9].

The COVID-19 pandemic led to a rapid shift to telemedicine services and had a significant negative impact on the health and well-being of children with NDDs [9]. For example, Zhang et al. [10] reported that children with ADHD had significantly worse behavioral symptoms and mood states, and parental stress increased during the pandemic compared to before. Althiabi [11] reported that the COVID-19 pandemic affected the mental health of parents with ASD children, which included loss of confidence and depression. This researcher also reported that anxiety states were significantly higher than before the COVID-19 pandemic.

Web-based interventions could address the issues mentioned above by providing an opportunity to receive effective interventions at a distance with less burden on therapists. Khan et al. [8] described web-based interventions as self-directed or therapist-assisted programs aiming at improving knowledge and providing support, care, and treatment. They used serious games and apps without physical activity for children with NDDs and showed a positive effect on reducing their symptoms. However, few systematic reviews have investigated the effects of web-based interventions that focus on exercise interventions in children with NDDs. The purpose of the present systematic review was to examine the effects of web-based exercise interventions on social skills, motor functions, and physical activity in children with NDDs.

## 2. Materials and Methods

### 2.1. Eligibility Criteria

This systematic review was conducted in accordance with the Preferred Reporting Items for Systematic Reviews and Meta-Analyses (PRISMA) statement [12]. The inclusion criteria were as follows: (a) patients with NDDs aged 18 years or younger; (b) include a web-based intervention; (c) include an intervention with exercise training; (d) published from 1994 to 2022; and (e) written in English. NDDs were selected with reference to the criteria of the Diagnostic and Statistical Manual of Mental Disorders, Fifth Edition (DSM-5) [2] or the International Statistical Classification of Diseases and Related Health Problems (ICD-10) [13]. The interventions included were home-based and web-based interventions offered through websites, apps, or social media. The exclusion criteria included (a) studies that were an observational study, review, meta-analysis, or study protocol, and (b) studies that did not include social skills, motor function, or physical activity as the outcomes.

### 2.2. Search Strategy

We searched the PubMed database for articles using different combinations of keywords as follows: #1 Neurodevelopmental Disorders [Title/Abstract], #2 NDDs [Title/Abstract], #3 Neurodevelopmental Disorders [MeSH Terms], #4 ASD [Title/Abstract], #5 Autism Spectrum Disorders [Title/Abstract], #6 Autistic Spectrum Disorders [Title/Abstract], #7 Autistic Disorders [Title/Abstract], #8 Asperger Syndrome [Title/Abstract], #9 Autism Spectrum Disorder [MeSH Terms], #10 ADHD [Title/Abstract], #11 Attention Deficit Disorder Hyperactivity [Title/Abstract], #12 Attention Deficit Disorder [Title/Abstract], #13 Attention Deficit Disorder with Hyperactivity [MeSH Terms], #14 LD [Title/Abstract], #15 Learning Disabilities [Title/Abstract], #16 Learning Disabilities [MeSH Terms], #17 Communication Disorders [Title/Abstract], #18 Communication Disorders [MeSH Terms], #19 Intellectual Disability [Title/Abstract], #20 Intellectual Disability [MeSH Terms], #21 Motor Skills Disorders [Title/Abstract], #22 Motor Skills Disorders [MeSH Terms], #23 Tic Disorders [Title/Abstract], #24 Tic Disorders [MeSH Terms], #25 Tourette Syndrome [Title/Abstract], #26 Developmental Coordination Disorder [Title/Abstract], #27 DCD [Title/Abstract], #28 [#1 or #2 or #3 or #4 or #5 or #6 or #7 or #8 or #9 or #10 or #11 or #12 or #13 or #14 or #15 or #16 or #17 or #18 or #19 or #20 or #21 or #22 or #23 or #24 or #25 or #26 or #27], #29 children [Title/Abstract], #30 adolescent [Title/Abstract], #31 youth [Title/Abstract], #32 preschooler [Title/Abstract], #33 preschool [Title/Abstract], #34 child, preschool [MeSH Terms], #35 adolescent [MeSH Terms], #36 child [Title/Abstract], #37 adolescents [Title/Abstract], #38 student [Title/Abstract], #39 students [Title/Abstract], #40 [#29 or #30 or #31 or #32 or #33 or #34 or #35 or #36 or #37 or #38 or #39], #41 Telerehabilitation [MeSH Terms], #42 telerehabilitation [Title/Abstract], #43 remote rehabilitation [Title/Abstract], #44 virtual rehabilitation [Title/Abstract], #45 Zoom [Title/Abstract], #46 WhatsApp [Title/Abstract], #47 Web-based [Title/Abstract], #48 home based [Title/Abstract], #49 [#41 or #42 or #43 or #44 or #45 or #46 or #47 or #48] #50 [#28 and #40 and #49]. The final search was conducted on 3 October 2022.

### 2.3. Selection Process

The screening process was performed by two independent evaluators (N.I. and A.O.). In case of a disagreement regarding eligibility, a third evaluator (R.Y.) performed the evaluation. During the first screening, we read the titles and abstracts of each study and selected relevant articles. During the second screening, we read the full text of the selected articles and excluded articles according to the exclusion criteria. We emailed the authors and other institutions and obtained four full-text files that were not available in our own institution.

### 2.4. Risk of Bias Assessment

Two researchers (N.I. and R.Y.) independently assessed the risk of bias in the included articles in accordance with the Cochrane Collaboration risk-of-bias tool [14] and integrated their results. Each of the seven domains (random sequence generation, allocation concealment, blinding [participants and personnel], blinding [outcome assessment], incomplete outcome data, selective reporting, and other source of bias) were evaluated as “low risk”, “unclear risk”, or “high risk”.

### 2.5. Data Collection Process

We extracted data regarding the target disease, sample size, age, female ratio, gender of participants, outcome measures, and intervention methods (frequency, time, duration, and type of exercise).

## 3. Results

### 3.1. Results of First and Second Screenings

**Figure 1:**
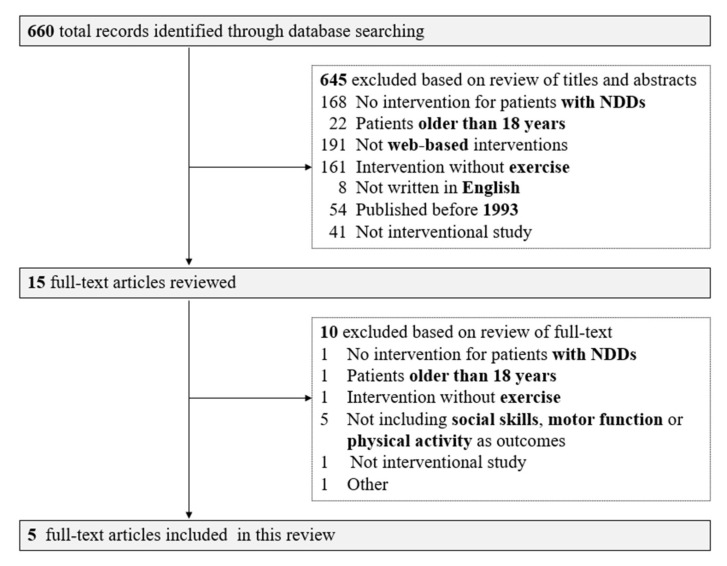
Flow diagram of this review.

We obtained 660 relevant articles from PubMed. During the first screening, 645 articles were excluded based on a review of the titles and abstracts. At the second screening, 10 more articles were excluded due to discrepancies in subjects, age, intervention methods, and outcome measures; finally, we included five full-text articles [15,16,17,18,19] in this review (Figure 1). Cohen’s Kappa coefficient for the screening performed by the two evaluators was 0.43 for the first screening and 0.61 for the second screening. Characteristics of the 5 included studies are summarized in Table 1.

In the five extracted papers, the intervention targets were ADHD in one paper [15], DCD in two papers [16,17], and ASD in two papers [18,19]. Each child was diagnosed according to the ICD-10 [13], DSM-5 [2], the Developmental Coordination Disorder Questionnaire (DCDQ) [20], the Childhood Autism Rating Scale (CARS) [21], the Modified Checklist for Autism in Toddlers (M-CHAT) [22], medical observations, or medical techniques. Diagnosis was made using the ICD-10 in one study [15], using the DSM-5 in one study [19], and using the DSM-5 and the DCDQ in two studies [16,17], and diagnosis was likely made using the CARS, the M-CHAT, medical observations, or medical technology in the remaining study [18], as the diagnostic method was not described. The participants ranged in age from 8 to 14 years, and the percentage of females ranged from 18.6% [15] to 52.4% [16,17].

Regarding the study design, one paper used RCTs [15], two papers used crossover RCTs [16,17], one paper was an exploratory mixed methods study [18], and one paper was a feasibility study without a control group [19]. The studies were conducted in Turkey in two papers [18,19], Australia in two papers [16,17], and Switzerland in one paper [15].

### 3.2. Exercise Intervention

Three studies [15,16,17] were based on interventions using video games, with one being offered via Zoom [18] and one being preferred via WhatsApp [19]. All of these interventions were designed to provide exercise. The active video games (AVGs) used in these interventions included Xbox Kinect (Microsoft, Redmond, WA, USA) [15], Xbox360 (Microsoft), PlayStation3 (Sony, Tokyo, Japan), Racket Sports (Ubisoft, Montreuil, France), and Cross Board 7 (Microsoft, Game Studios, USA) [16,17]. The popular games were Kinect Sports (Microsoft), TV Superstars (Sony), and Dance Central (Microsoft) [16]. The exercise intervention using Zoom [18] included home-based exercises, dance activities, and fitness activities. The WhatsApp-based exercise intervention [19] included home-based exercises, fun games, dance, daily housework, meditation, and fitness activities.

To implement physical activity, all studies involved the participation of professionals in either pre-intervention explanations, during the intervention, or in post-intervention follow-up. In only one study, the intervention was conducted by a trainer each time [18]. Parents received the intervention with their children under the guidance of the trainer. In one study where each child was monitored during the first intervention by a trainer [15], parents were instructed to provide assistance and support during the intervention. In one study in which parental guidance was the most substantial [19], the definitions of exercise and information were provided to parents in advance via a YouTube video, and the parents were instructed to participate in the exercise together with their children. Two studies [16,17] did not require monitoring during the intervention. The duration of the interventions ranged from 4 [19] to 16 weeks [16,17].

### 3.3. Motor Function

Two of the five studies measured motor function using the German Motor Test [15] and the Movement Assessment Battery for Children–Second Edition (MABC-2) [16,17] as an outcome measure. The German Motor Test measures basic motor skills, such as endurance, speed, muscle strength, coordination, and flexibility [23]. The MABC-2 assesses motor coordination and consists of eight tasks in the three categories of manual dexterity, aiming and catching, and balance [24]. There were significant improvements in motor function on the single test items of the German Motor Test in lateral jumping (F(2, 48) = 4.49, *p* = 0.039, Cohen’s d = 0.61) and push-ups (F(2, 48) = 4.73, *p* = 0.035, d = 0.63) after an AVG intervention was provided for children with ADHD [15]. In contrast, there was no significant improvement in children with DCD [16,17].

### 3.4. Executive Function

One of the five studies measured executive function (EF) [15] using the E-Prime Software. In this article, the E-Prime Software was used as a computer-based EF test to measure core EFs, such as inhibition, switching, and updating. The exergaming intervention for children with ADHD showed a significant improvement in executive function with regard to reaction times in inhibition (F(2, 48) = 4.08, *p* = 0.049, d = 0.58) and switching (F(2, 48) = 5.09, *p* = 0.029, d = 0.65) [15].

### 3.5. Physical Activity

Three of the five studies used physical activity as an outcome measure [16,18,19], as determined by the MABC-2 [16], accelerometers [16], and the Leisure Time Exercise Questionnaire (LTEQ) [18,19]. A study with children with ASD using Zoom [18] showed a statistically significant increase in physical activity (F = 95.396, *p* = 0.000, Ƞ2 = 0.834). Another study with children with ASD using WhatsApp [19] also showed a statistically significant increase in physical activity (d = 2.80; d > 1.00). A study investigating an intervention using AVGs in children with DCD showed no significant increase in physical activity [16].

### 3.6. Risk of Bias in the Studies

**Figure 2:**
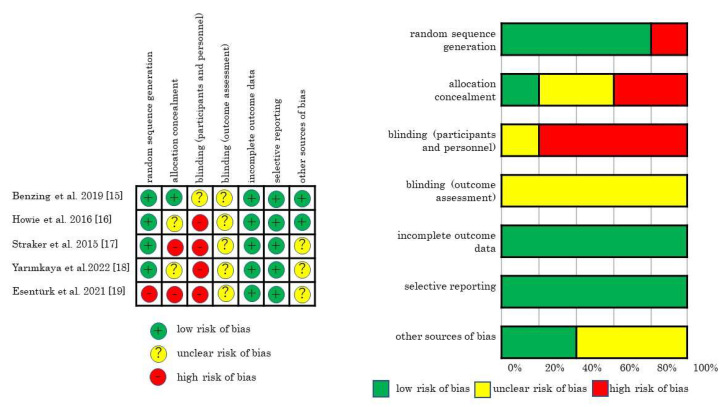
The risk of bias of each study and domain.

A summary of the risk of bias for each study and domain is shown in Figure 2. All articles showed a low risk of bias in terms of incomplete outcome data and selective reporting and an unclear risk of bias in terms of blinding outcome assessment. Most of the articles showed a high risk of bias in terms of blinding participants and personnel.

## 4. Discussion

### 4.1. Brief Summary of This Review

We systematically reviewed the effects of web-based exercise interventions for children with NDDs. The present review included three studies using AVG interventions [15,16,17], one study using a Zoom-based intervention [18], and one study using a WhatsApp-based intervention [19]. Three of the five studies showed improvements in motor function, executive function, or physical activity.

### 4.2. Comparison with Previous Studies

A systematic review and meta-analysis by Khan et al. [8] found that web-based interventions without exercise were effective in reducing the symptoms of NDDs. The interventions in their review included apps, serious games, videoconferencing, and virtual environments. The participants had ASD, tic disorders, ADHD, learning disabilities, and computational disabilities. The present review focused on interventions that were exercise interventions using AVGs and videoconferencing, and the three disorders targeted were ASD, ADHD, and DCD. The present review found that web-based exercise interventions might improve motor function, executive function, and physical activity, supporting the findings of the previous review [8].

### 4.3. Possible Explanations and Implications

The web-based exercise interventions for children with ASD [18,19] and ADHD [15] were effective in improving executive function, motor function, and physical activity, whereas those for children with DCD [16,17] were not effective in improving physical activity and motor coordination. These differences might have resulted from the type of disorders, the intervention methods, parental and professional participation in the intervention, or the instructional methods.

Two studies [16,17] found no improvement in motor coordination or physical activity after using a home-based AVG intervention in children with DCD. However, Jelsma et al. [25] specifically targeted an intervention using the Wii Fit balance board to DCD children with balance deficits. This resulted in a significant improvement in scores on the balance component of the MABC-2. Therefore, the differences in the results might not relate to the differences in the type of disorders.

Using an AVG intervention, one study with children with ADHD [15] showed improvement in executive and motor function, whereas two other studies with children with DCD [16,17] showed no improvement in either physical activity or motor coordination. One of the differences was in the content of the games used in the intervention: the study with children with ADHD [15] used predetermined games, whereas the studies with children with DCD [16,17] allowed the participants to choose from a variety of non-violent games. Children with DCD are known to avoid activities that challenge their motor impairments [26]. Therefore, the participants’ preference for games might have prevented them from trying games that might involve motor coordination and high physical activity. In addition, the study by Jelsma et al. [25] focused only on balance and resulted in an improvement in symptoms. In the studies included in the present review [16,17], the interventions were aimed at improving various aspects of motor coordination, which might have resulted in a lack of improvement in the symptoms.

A study with children with ASD using a Zoom-based intervention [18] showed an improvement in physical activity as a result of the constant synchronization with a trainer during the intervention. A study with children with ASD using a WhatsApp-based intervention [19] also showed an improvement in physical activity as a result of providing information to parents in advance and requiring parents to participate in the exercise. The intervention using AVGs for children with ADHD [15] required initial instruction by specially trained research assistants and parental support during training. From these results, appropriate assistance and support to parents and children by experts may lead to effective interventions. In addition, the intervention using AVGs for children with DCD [16,17] could be implemented without parental supervision. Therefore, this study might not have provided an exercise intervention with sufficient quality to improve physical activity or motor function.

### 4.4. Strengths and Limitations

To the best of our knowledge, this is the first systematic review of the effects of web-based interventions, specifically exercise training interventions, for children with NDDs. The interventions were examined based on frequency, time, type, and duration of training, as well as the presence or absence of support by parents or instructors.

The present review has some limitations, however. First, because we only used PubMed as the database, the number of studies and samples included in the present review was very small and limited. Second, because the outcome measures and their units differed, a meta-analysis could not be performed. Third, the included studies are different in terms of study design, intervention methods (i.e., frequency, time, duration, and type of exercise), and monitoring during intervention. Fourth, children with DCD have different clinical characteristics from children with ASD and ADHD, even though, as stated by the authors, many patients with NIDDS have two or more comorbid disorders, and motor dysfunction is often comorbid with ASD. However, we could not investigate these points in the present review. Thus, we need to conduct more research work in future trials. Despite the limitations of these few studies, this review showing the effects of web-based interventions for children with NDDs has the potential to be of great use in future research and intervention development.

## 5. Conclusions

Web-based exercise interventions for children with ASD and ADHD may improve their motor function, executive function, and physical activity, but they may not be effective for children with NDDs. These interventions may be more effective when the content of the intervention is based on objectives and symptoms, guidance is provided by specialists, or sufficient explanation and support are provided to parents. However, more research is needed to statistically evaluate the effectiveness of this type of interventions.

## Figures and Tables

**Table 1:** Summary of articles.

Study	Study Design	Disease	Sample Size	Age (Years)	Female (%)	Outcome Measures	Frequency (/Week)	Time	Duration (Weeks)	Type	Results
Benzing et al. 2019 [15]	RCT	ADHD	51	8–12	18.6	E-Prime Software, Conners-3 scales, DSM-Ⅳ-TR symptom scales, German Motor Test	3	10 min/day	8	Xbox Kinect	Exergames significantly improved EFs, general psychopathology, and motor abilities.
Howie et al. 2016 [16]	Crossover RCT	DCD	21	9–12	52.4	MABC-2, accelerometers	4–5	140 min/week	16	PlayStation 3, Xbox360, non-violent games	There were no differences in PA after the AVG intervention.
Straker et al. 2015 [17]	Crossover RCT	DCD	21	9–12	52.4	MABC-2, accelerometers	4–5	140 min/week	16	PlayStation3, Xbox360, non-violent games	There were no differences in motor coordination during an intervention with AVGs.
Yarımkaya et al. 2022 [18]	Explanatory mixed methods design	ASD	22	10.7 (average)	31.8	LTEQ, semi-structured interview questions	4	30–35 min/day	10	Home-based exercise, dance activities, fitness activities (Zoom)	PA significantly increased through Zoom-delivered physical activity compared to the control group.
Esentürk et al. 2021 [19]	Feasibility study	ASD	14	9–14	42.9	Feasibility questionnaire, LTEQ	7	20–30 min/day	4	Home-based exercise, fun games, dance, housework, meditation, fitness activities (WhatsApp)	PA was positively affected after the WhatsApp-based intervention.

ADHD, attention deficit hyperactivity disorder; ASD, autism spectrum disorder; AVGs, active video games; DCD, developmental coordination disorder; EF, executive function; LTEQ, Leisure Time Exercise Questionnaire; MABC-2, Movement Assessment Battery for Children–Second Edition; PA, physical activity; RCT, randomized controlled trial.

## Data Availability

The data underlying this article cannot be shared publicly due to privacy concerns of individuals who participated in the studies. The data will be shared by the corresponding author upon reasonable request.
